# Integrated Multi-Omics Analysis Reveals Key Metabolites and Regulatory Networks Underlying Flower Color Variation in F1 Hybrids of *Cymbidium eburneum* × *Cymbidium insigne*

**DOI:** 10.3390/genes17091003

**Published:** 2026-08-26

**Authors:** Yu Han, Yutong Cui, Yu Chen, Dandan Rao, Erhuan Wu, Rongcun Gan, Tengmin Li

**Affiliations:** 1 Forestry High-Tech Research Institute, Hainan Academy of Forestry (Hainan Academy of Mangrove), Haikou 571100, China; 2 The Innovation Platform for Academicians of Hainan Province, Haikou 571100, China

**Keywords:** flower color, transcriptomics, metabolomics, WGCNA, correlation analysis, *Cymbidium*, anthocyanin, flavonoid biosynthesis

## Abstract

**Background/Objectives:** Flower color is a core ornamental trait in orchids (*Orchidaceae*), arising from the biosynthesis, accumulation, and spatial distribution of pigments under multilayered transcriptional regulation. **Methods:** We performed parallel RNA-seq transcriptomic and widely targeted metabolomic profiling of 15 samples representing five floral tissue/color categories from an F1 population of *Cymbidium eburneum* (‘Duzhan Chun’) × *Cymbidium insigne* (‘Meihua Lan’). **Results:** Across four prespecified pairwise comparisons, 4891 non-redundant differentially expressed genes (DEGs) and 383 non-redundant differentially accumulated metabolites (DAMs) were identified. WGCNA resolved 12 co-expression modules. Recalculation from archived files identified darkseagreen4 as the module most positively associated with red tissues (r = 0.888), lightgreen as the module most positively associated with yellow tissues (r = 0.916), and plum1 as the module most strongly associated with white tissues in the negative direction (r = −0.983). Genes meeting |MM| ≥ 0.80, |GS| ≥ 0.80, MM *p* < 0.01, and GS *p* < 0.01 were retained as color-associated candidates. Anthocyanin derivatives including Cyanidin-3-O-sambubioside-5-O-glucoside, Delphinidin-3-rutinoside, and Peonidin-3,5-O-di-β-glucoside were associated with red floral tissues. **Conclusions:** Integrated analyses consistently highlighted phenylpropanoid and flavonoid biosynthesis, whereas isoquinoline alkaloid enrichment remains hypothesis-generating. These associations nominate candidates but do not establish causal pigmentation regulators.

## 1. Introduction

*Orchidaceae* is one of the most species-rich families of flowering plants, renowned for its remarkable diversity in floral architecture, longevity, and color [1,2]. Flower color, as a primary driver of ornamental value, arises from the interaction of three major classes of pigments-flavonoids (including anthocyanins, flavones, and flavonols), carotenoids, and betalains-which differ in type, concentration, cellular distribution, and behavior within the vacuolar microenvironment [3,4]. Among these, anthocyanins are primarily responsible for red, pink, purple, and blue hues; carotenoids govern yellow, orange, and red tones; while the absence of pigments results in white flowers, with specific shades potentially modulated by cell structure or colorless flavonoids such as flavonols [5,6].

At the molecular level, flower color is governed by a highly conserved and precisely regulated metabolic and transcriptional network. Anthocyanin biosynthesis begins with phenylalanine and proceeds through the phenylpropanoid pathway via sequential catalysis by key enzymes including phenylalanine ammonia-lyase (PAL), chalcone synthase (CHS), chalcone isomerase (CHI), flavanone 3-hydroxylase (F3H), flavonoid 3′-hydroxylase (F3′H), flavonoid 3′,5′-hydroxylase (F3′5′H), dihydroflavonol 4-reductase (DFR), anthocyanidin synthase (ANS), and UDP-glucose:flavonoid 3-O-glucosyltransferase (UFGT), culminating in pigmented anthocyanin glycosides stored in vacuoles [7,8,9,10]. Subcellular compartmentalization is also critical, with glutathione S-transferases (GSTs) and transporters (MATE, ABC families) responsible for shuttling anthocyanins into vacuoles [11,12]. Transcriptional regulation of the pathway is predominantly mediated by a conserved MBW ternary complex comprising R2R3-MYB, bHLH, and WD40 transcription factors, which collectively activate or suppress downstream structural gene expression [13,14]. This regulatory paradigm has been well documented in *Rosa rugosa* [15], *Petunia hybrida* [16], *Phalaenopsis* [17], and *Oncidium* [18].

Integrated transcriptomics and metabolomics have emerged as a powerful strategy for dissecting the molecular basis of complex biological traits at a systems level [19,20]. By correlating gene expression profiles with metabolite accumulation patterns, this approach efficiently identifies key regulatory genes and metabolites and generates testable gene–metabolite association networks. Notable examples include the identification of RhMYB114 and RhbHLH42 as core regulators of anthocyanin biosynthesis in *R. rugosa* petals [15], as well as studies in *Paeonia* [21], *Lilium* [22], *Chrysanthemum* [23], and *Gerbera* [24].

Despite this progress, the genome-wide regulatory landscape of flower color in orchids-particularly in interspecific hybrids exhibiting extensive color variation-remains incompletely understood. *Cymbidium eburneum* (‘Duzhan Chun’) and *Cymbidium insigne* (‘Meihua Lan’) represent contrasting parental phenotypes, with *C. eburneum* bearing ivory-white flowers and *C. insigne* displaying deep purple-brown flowers with dense veining. Their F1 hybrid progeny exhibit broad continuous color variation spanning both parental extremes and numerous intermediary phenotypes [25], providing an ideal system for investigating the molecular basis of floral pigmentation with reduced genetic background noise.

In the present study, we employed widely targeted metabolomics and high-throughput RNA-seq transcriptomics on five F1 individuals of *C. eburneum* × *C. insigne* representing distinct flower colors (white, yellow, and red). Our objectives were: (1) to comprehensively characterize the metabolite basis of flower color differentiation and identify key chromogenic compounds; (2) to identify DEGs and transcription factors at the genome-wide level; and (3) to construct gene–metabolite–phenotype regulatory networks using WGCNA and integrated correlation analysis. This study represents the first systems-level characterization of flower color formation in *Cymbidium* interspecific hybrid offspring, providing valuable genetic and metabolic resources for molecular orchid breeding.

## 2. Materials and Methods

### 2.1. Plant Materials

In 2015, wild *C. eburneum* (‘Duzhan Chun’) and *C. insigne* (‘Meihua Lan’) were hybridized. Hybrid seeds were obtained in March 2017 and sown to generate an F1 population maintained at the Orchid Germplasm Resource Nursery of the Yunlong Base, Hainan Academy of Forestry Sciences (19°52′21″ N, 110°28′59″ E). Between December 2023 and March 2024, healthy F1 individuals free of visible pests and disease were sampled at peak flowering. Flower-color phenotyping was conducted in accordance with the 2020 forestry industry standard LY/T 3206–2020, and colors were assigned using the Royal Horticultural Society (RHS) Colour Chart. Five tissue/color categories (F1-EG, F1-ER, F1-EW, F2-CR, and F2-CW) were examined. For each category, 15 plants showing the designated tissue/color phenotype were selected. One flower of comparable developmental stage and without visible damage was collected from each plant. The 15 flowers were assigned to three non-overlapping biological replicate pools, with each pool containing five flowers obtained from five different plants. No plant or flower contributed to more than one replicate; thus, the three replicates for a category represented three independently collected sets of five plants and were not generated by subdividing a single pooled sample. The designated floral tissue was excised independently from each flower. Red or white labellum tissue was collected for F1-ER and F1-EW, whereas petal and sepal tissues were collected for F1-EG, F2-CR, and F2-CW. Equal amounts of the designated tissue from the five plants within each set were combined to form one biological replicate. Each replicate pool was labeled, snap-frozen in liquid nitrogen for 20 min, and stored separately at −80 °C. The three replicate pools were independently homogenized and processed throughout sample preparation. Aliquots from each independently homogenized replicate were then used for RNA-seq and metabolomic profiling. Accordingly, each category comprised three independent pooled biological replicates representing 15 distinct plants, yielding 15 replicate samples across the five categories. Representative parental and offspring flower phenotypes and the category codes are shown in Figure S1. The five sample IDs, tissues, colors, and Royal Horticultural Society (RHS) color codes are defined in Table 1.

### 2.2. Widely Targeted Metabolomic Analysis

Metabolite extraction was performed following the method of Weckwerth [26] with modifications. Approximately 50 mg of frozen petal powder was weighed and extracted with 1.0 mL of pre-cooled 70% methanol in water (containing internal standards), vortexed, and ultrasonicated at 4 °C for 30 min. Extracts were centrifuged at 12,000 rpm for 10 min, and the supernatant was filtered through a 0.22 μm membrane filter prior to UPLC-MS/MS analysis. The injection volume was 2 μL. Chromatographic separation was performed on a Waters ACQUITY UPLC HSS T3 column (Waters Corporation, Milford, MA, USA) (2.1 mm × 100 mm, 1.8 μm) at 40 °C, using 0.1% formic acid in water (mobile phase A) and 0.1% formic acid in acetonitrile (mobile phase B) with a gradient elution program. Mass spectrometric detection was performed on an SCIEX QTRAP 6500+ system (SCIEX, Framingham, MA, USA) equipped with a TurboIonSpray electrospray ionization source and operated in positive and negative ion modes using multiple reaction monitoring (MRM). The source temperature was 500 °C; the ion-spray voltage was +5500 V in positive mode and −4500 V in negative mode. Ion-source gas 1 (GS1), ion-source gas 2 (GS2), and curtain gas (CUR) were set to 55, 60, and 25 psi, respectively, and the collision gas (CAD) was set to high. Declustering potential and collision energy were optimized individually for each MRM transition. Metabolites were identified against an in-house database and public databases (MassBank, HMDB). Peak area integration and relative quantification were performed using OSI-SMMS software. Multivariate statistical analyses-including unsupervised principal component analysis (PCA) and supervised orthogonal partial least squares discriminant analysis (OPLS-DA)-were conducted using SIMCA-P 14.1. Differentially accumulated metabolites (DAMs) were filtered using OPLS-DA variable importance in projection (VIP) > 1.5, Student’s *t*-test (*p* < 0.05), and fold change (FC) > 2 or <0.5 as criteria [8]. Additionally, K-means clustering was applied to DAMs to classify expression patterns and each cluster was subjected to KEGG pathway enrichment analysis.

### 2.3. Transcriptomic Sequencing and Analysis

Total RNA was extracted using a plant total RNA extraction kit (Omega Bio-tek, Norcross, GA, USA). RNA quality was assessed with an Agilent 2100 Bioanalyzer (Agilent Technologies, Santa Clara, CA, USA), and all samples had RNA integrity number values ≥ 8.0. This study analyzed 15 libraries from five selected tissue/color categories (F1_EG, F1_ER, F1_EW, F2_CR, and F2_CW), with three samples per category. Libraries were sequenced on an Illumina platform (Illumina, San Diego, CA, USA) using paired-end reads. The raw RNA-seq data have been deposited in the NCBI Sequence Read Archive under BioProject accession PRJNA1338804. According to the project completion report, paired-end raw reads were quality-filtered with fastp v0.23.2 within the nf-core RNA-seq workflow [27]. Adapter trimming by paired-end overlap detection and quality/length filtering were applied using the following settings: qualified-quality Phred threshold, 15; maximum proportion of bases below this threshold, 40%; maximum number of ambiguous N bases per read, 5; and minimum retained read length, 15 nt. Poly-G tail trimming and paired-end adapter detection were enabled when applicable. Clean reads were aligned with STAR [28] to the publicly available annotated *Cymbidium ensifolium* (Jianlan) reference genome deposited in the Genome Warehouse (GWH), National Genomics Data Center, under assembly accession GWHBCII00000000 (BioProject PRJCA005355; BioSample SAMC383359; https://ngdc.cncb.ac.cn/gwh/Assembly/20686, accessed on 20 August 2026). This *C. ensifolium* genomic resource was described by Ai et al. [29]. The assembly was generated from PacBio reads at 98× coverage using wtdbg v1.2.8 and released as a draft genome at the scaffold level. It spans 3,623,198,571 bp (3.62 Gb), with a GC content of 33.58%, 26,303 scaffolds, a scaffold N50 of 92,663,771 bp (92.66 Mb), a scaffold N90 of 53,000 bp, a maximum scaffold length of 208,335,477 bp, an average scaffold length of 137,748 bp, a minimum scaffold length of 201 bp, and 28,738 annotated protein-coding genes. Alignment statistics were summarized using Samtools and MultiQC. Salmon was used to obtain gene-level read counts, whereas StringTie [30] was used to estimate FPKM values for expression visualization. Differential expression was performed in R 4.3 using SARTools with DESeq2 v1.42.0. Raw counts were normalized using the DESeq2 median-of-ratios method, differential expression was assessed with the Wald test, and *p*-values were adjusted by the Benjamini–Hochberg procedure. DEGs were defined by FDR < 0.05 and |log2 fold change| > 1. GO and KEGG enrichment analyses were conducted using the procedures described in the project workflow and visualized with clusterProfiler [31].

### 2.4. Weighted Gene Co-Expression Network Analysis (WGCNA)

Weighted gene co-expression network analysis was performed in R using the WGCNA package [32] on the expression matrix of the 15 RNA-seq samples. Genes and samples failing expression- and quality-control filters were removed, and the archived analysis resolved 12 merged co-expression modules. Because the supplied WGCNA result package contains module assignments, module eigengenes, and color-specific MM/GS statistics but does not contain a soft-threshold selection plot, candidate-power table, analysis script, or execution log, no numerical soft-thresholding power is reported here. Module–color correlations were recalculated from the archived module eigengenes and independently checked from the relationship between module membership and color-specific gene significance. Module membership (MM) was defined as the Pearson correlation between a gene and its module eigengene, and gene significance (GS) as the Pearson correlation between gene expression and the red, yellow, or white phenotype indicator. Candidate genes were required to satisfy |MM| ≥ 0.80, |GS| ≥ 0.80, MM *p* < 0.01, and GS *p* < 0.01, and were ranked by |MM × GS|. Because the analysis included 15 samples and the floral organ was partly confounded with color category, all WGCNA results were interpreted as exploratory associations.

### 2.5. Integrated Transcriptome–Metabolome Correlation Analysis

Gene expression data from key WGCNA modules were correlated with the relative abundance of all differentially accumulated flavonoid metabolites by Pearson correlation analysis. Gene–metabolite pairs with |r| > 0.8 and *p* < 0.001 were considered significantly correlated. Based on KEGG pathway annotation, significantly correlated gene–metabolite pairs located within the flavonoid biosynthesis pathway were selected and visualized as a regulatory network using Cytoscape v3.9.1 [33].

### 2.6. Quantitative Real-Time PCR (qRT-PCR) Validation

To compare the archived RT-qPCR profiles with the RNA-seq expression trends, six differentially expressed genes (DEGs), encompassing structural biosynthesis genes and transcription factors, were selected for quantitative real-time PCR (qRT-PCR). First-strand cDNA was synthesized using the PrimeScript RT Reagent Kit with gDNA Eraser (Takara Bio Inc., Kusatsu, Shiga, Japan). Reactions were performed using the SYBR Green Premix Pro Taq HS qPCR Kit (Accurate Biotechnology (Hunan) Co., Ltd., Changsha, China) on a QuantStudio 5 real-time PCR system (Applied Biosystems, Foster City, CA, USA). *CymTIP41* and *CymEF1α* were used as reference genes [18], and relative expression was calculated using the 2^−ΔΔCt^ method. The archived experimental description records three technical replicates per sample; however, the replicate-level Ct records are no longer available for re-analysis. Consequently, replicate dispersion and inferential statistics could not be calculated from the retained records. Primer sequences and amplicon characteristics are listed in Table 2.

## 3. Results

### 3.1. Metabolomic Profiling and Differentially Accumulated Metabolites

Because tissue identity differed among categories, F1-ER versus F1-EW (labellum versus labellum) and F2-CR versus F2-CW (petal + sepal versus petal + sepal) were treated as the primary tissue-matched color contrasts. F1-EG versus F1-EW and F1-ER versus F1-EG compare different floral organs and were retained only as exploratory tissue/color-category contrasts; their DEGs were not considered to be associated with the production of specific effects on color.

**Figure 1:**
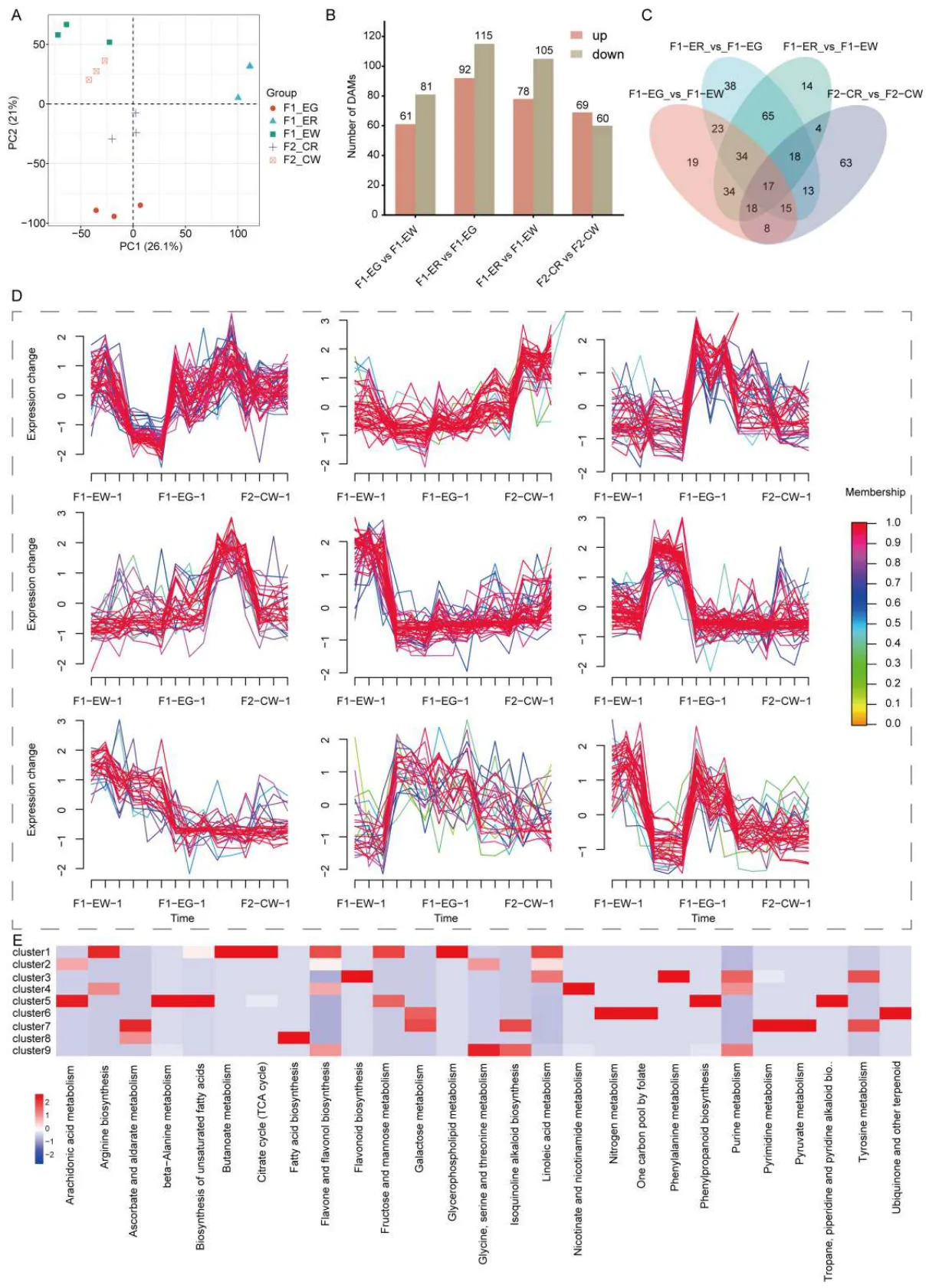
Metabolomic profiles of 15 samples representing five tissue/color categories. (**A**) Principal component analysis (PCA); points are individual biological replicate pools and point colors identify F1-EG, F1-ER, F1-EW, F2-CR, and F2-CW as defined in Table 1. (**B**) Numbers of differentially accumulated metabolites (DAMs) in four comparisons; “up” and “down” denote higher and lower accumulation, respectively, in the first category named relative to the second. (**C**) Venn diagram of 383 non-redundant DAMs, including 17 shared by all comparisons. (**D**) Nine archived K-means panels corresponding to clusters 1–9; each line represents one DAM, the *y*-axis shows standardized expression change, and line color follows the displayed cluster-membership scale (0–1). The archived panel contains F1-EW-1, F1-EG-1, and F2-CW-1 only; “-1” is a sample-identifier suffix denoting the first archived sample and is not a negative value. F1-ER and F2-CR were absent from this exported clustering panel. Therefore, panels (**D**,**E**) are interpreted descriptively for this subset and not as five-category evidence. (**E**) KEGG pathway profiles for clusters 1–9; red indicates relatively higher and blue relatively lower standardized values according to the displayed scale. Three biological replicate pools were analyzed per tissue/color category in panels (**A**–**C**).

To characterize metabolites associated with the five selected tissue/color categories, widely targeted metabolomics was performed on the same 15 samples used for transcriptomic analysis. PCA separated the five categories and showed clustering of the three samples within each category (Figure 1A). The four pairwise comparisons contained 142 DAMs (61 up-regulated and 81 down-regulated) in F1-EG versus F1-EW, 207 DAMs (92 up and 115 down) in F1-ER versus F1-EG, 183 DAMs (78 up and 105 down) in F1-ER versus F1-EW, and 129 DAMs (69 up and 60 down) in F2-CR versus F2-CW (Figure 1B). These counts sum to 661 comparison-specific occurrences; Venn analysis showed that their non-redundant union comprised 383 DAMs, including 17 shared by all four comparisons (Figure 1C).

KEGG enrichment analysis of DAMs from each comparison highlighted several pathways. In F1-EG vs. F1-EW, phenylpropanoid biosynthesis was the most significantly enriched pathway. In F1-ER vs. F1-EG, tyrosine metabolism and galactose metabolism were prominently enriched. In F1-ER vs. F1-EW, phenylpropanoid biosynthesis and flavonoid biosynthesis were the top enriched pathways (Figure S2). In F2-CR vs. F2-CW, biosynthesis of amino acids and phenylpropanoid biosynthesis were most significantly represented. The archived K-means output contained nine clusters for the three-sample subset displayed in Figure 1D,E; because two categories were absent from that export, these panels were not used for five-category biological inference.

### 3.2. Transcriptomic Profiling and Differentially Expressed Genes

**Figure 2:**
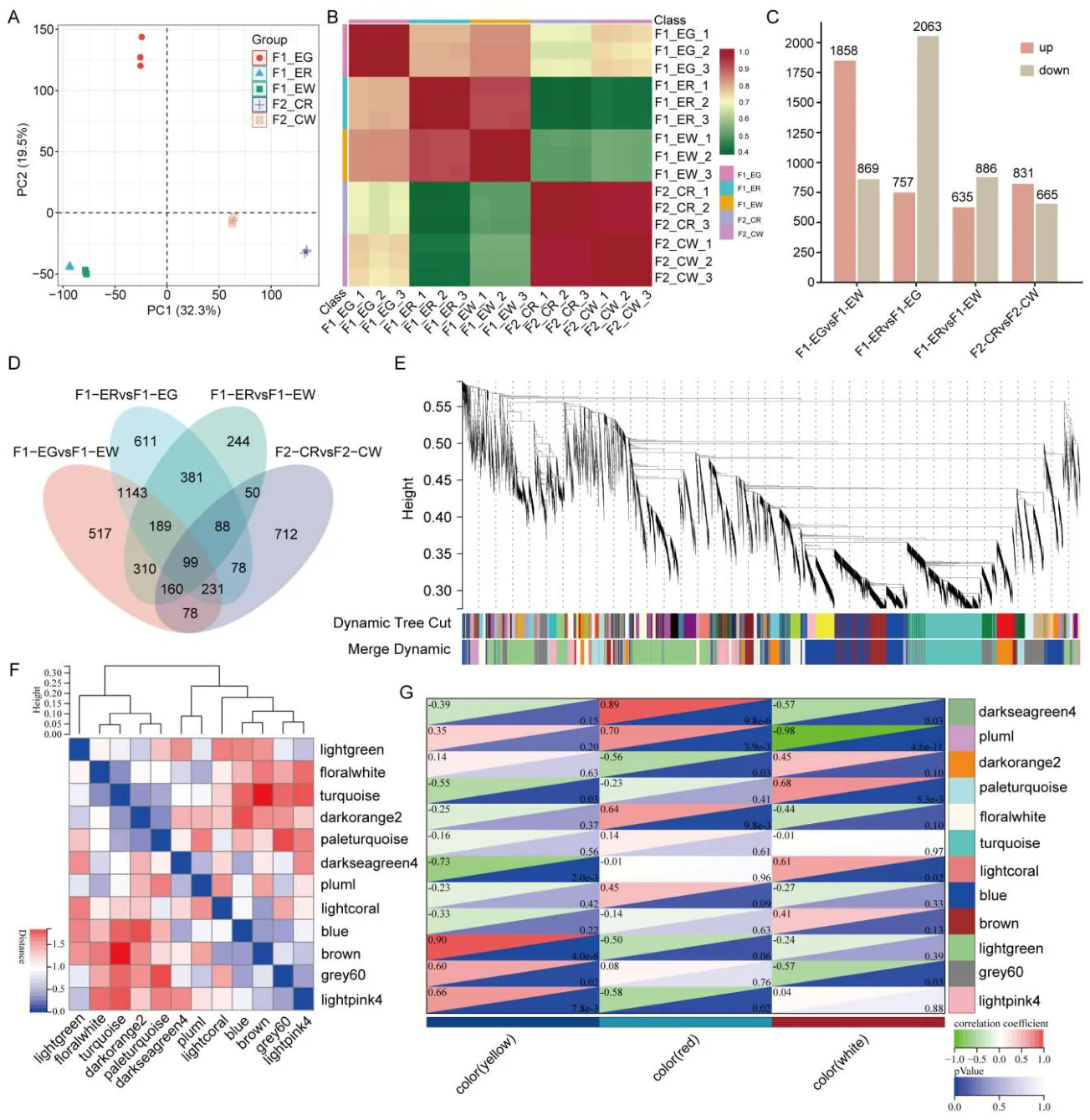
Transcriptomic analysis and exploratory weighted gene co-expression network analysis (WGCNA) of 15 RNA-seq libraries. (**A**) PCA of individual libraries; colors identify the five sample categories defined in Table 1. (**B**) Pearson sample-correlation heatmap; warmer colors indicate higher positive correlation and cooler/green colors lower correlation according to the scale. (**C**) Numbers of up- and down-regulated differentially expressed genes (DEGs) in four pairwise comparisons; up/down are defined relative to the first category named. (**D**) Venn diagram of unique and shared DEGs. (**E**) Hierarchical gene-clustering dendrogram; the colored bands show dynamic-tree-cut and merged WGCNA module assignments. (**F**) Module-eigengene expression heatmap; rows are modules, columns are samples, red denotes relatively higher and blue relatively lower standardized eigengene expression. (**G**) Module–trait correlations; the three columns are the yellow, red, and white phenotype indicators. Labels such as blue, turquoise, and red along the rows are WGCNA module names rather than phenotype categories. The correlation-coefficient and *p*-value color scales are defined by the two legends at the right, and each cell reports both values. Twelve merged modules were recovered from archived WGCNA outputs. A numerical soft-threshold power is not shown because the supplied package did not contain the diagnostic output or execution record.

RNA-seq was performed on 15 samples (five color categories × three biological replicates), yielding a total of 177.48 Gb of raw data. Each sample generated ≥ 8.37 Gb of clean data, with Q30 base percentages ≥ 90.41% and mapping rates to the reference genome ≥ 87.20%. PCA and Pearson correlation heat maps showed clustering among the reported replicates (Figure 2A,B).

Differential expression analysis identified 2727 DEGs (1858 up-, 869 down-regulated) in F1-EG vs. F1-EW; 2820 DEGs (757 up-, 2063 down-regulated) in F1-ER vs. F1-EG; 1521 DEGs (635 up-, 886 down-regulated) in F1-ER vs. F1-EW; and 1496 DEGs (831 up-, 665 down-regulated) in F2-CR vs. F2-CW (Figure 2C). Venn diagram analysis yielded 4891 non-redundant DEGs across the union of the four contrasts, including 99 DEGs shared by all four comparisons (Figure 2D).

GO enrichment analysis indicated that DEGs in F1-EG vs. F1-EW were enriched in protein domain-specific binding, demethylation, and salicylic acid binding; DEGs in F1-ER vs. F1-EG were enriched in chloroplast thylakoid membrane and DNA photolyase activity terms; DEGs in F1-ER vs. F1-EW were significantly enriched in chalcone isomerase activity, flavonoid biosynthetic process, and regulation of transcription; DEGs in F2-CR vs. F2-CW were enriched in pinoresinol reductase activity and response to light (Figure S3). KEGG analysis revealed that phenylpropanoid biosynthesis and flavonoid biosynthesis were consistently among the most significantly enriched pathways across comparisons, underscoring their central roles in flower color differentiation (Figure S4).

### 3.3. WGCNA Identifies Flower Color-Associated Co-Expression Modules

Re-analysis of the archived WGCNA result files identified 12 merged co-expression modules (Figure 2E,F). The darkseagreen4 module showed the strongest positive association with red tissues (r = 0.888), and 61 genes in this module met the prespecified MM/GS thresholds. The red-associated candidate genes listed in Table 3 were *JL016799*, *JL020957*, *JL000049*, *JL010156*, *JL017507*, *JL027414*, *JL007156*, *JL000779*, *JL010027*, and *JL014470*. The lightgreen module showed the strongest positive association with yellow tissues (r = 0.916); 737 genes met the thresholds, and the yellow-associated candidate genes listed in Table 3 were *JL020768*, *JL010806*, *JL010805*, *JL001469*, *JL010486*, *JL016806*, *JL009410*, *JL002831*, *JL012705*, and *JL011521*. For white tissues, plum1 showed the strongest absolute association, which was negative (r = −0.983). The white-associated candidate genes listed in Table 3 were *JL015236*, *JL019281*, *JL001887*, *JL020762*, *JL003788*, *JL009407*, *JL016580*, *JL020046*, *JL019159*, and *JL004250*. The negative plum1–white association indicates that the module eigengene and many positively loaded module genes decreased with the white phenotype; it does not define a white-pigment biosynthetic module. Candidate ranking reflects co-expression and phenotype association only, not demonstrated regulatory function.

### 3.4. Integrated Transcriptome–Metabolome Analysis

**Figure 3:**
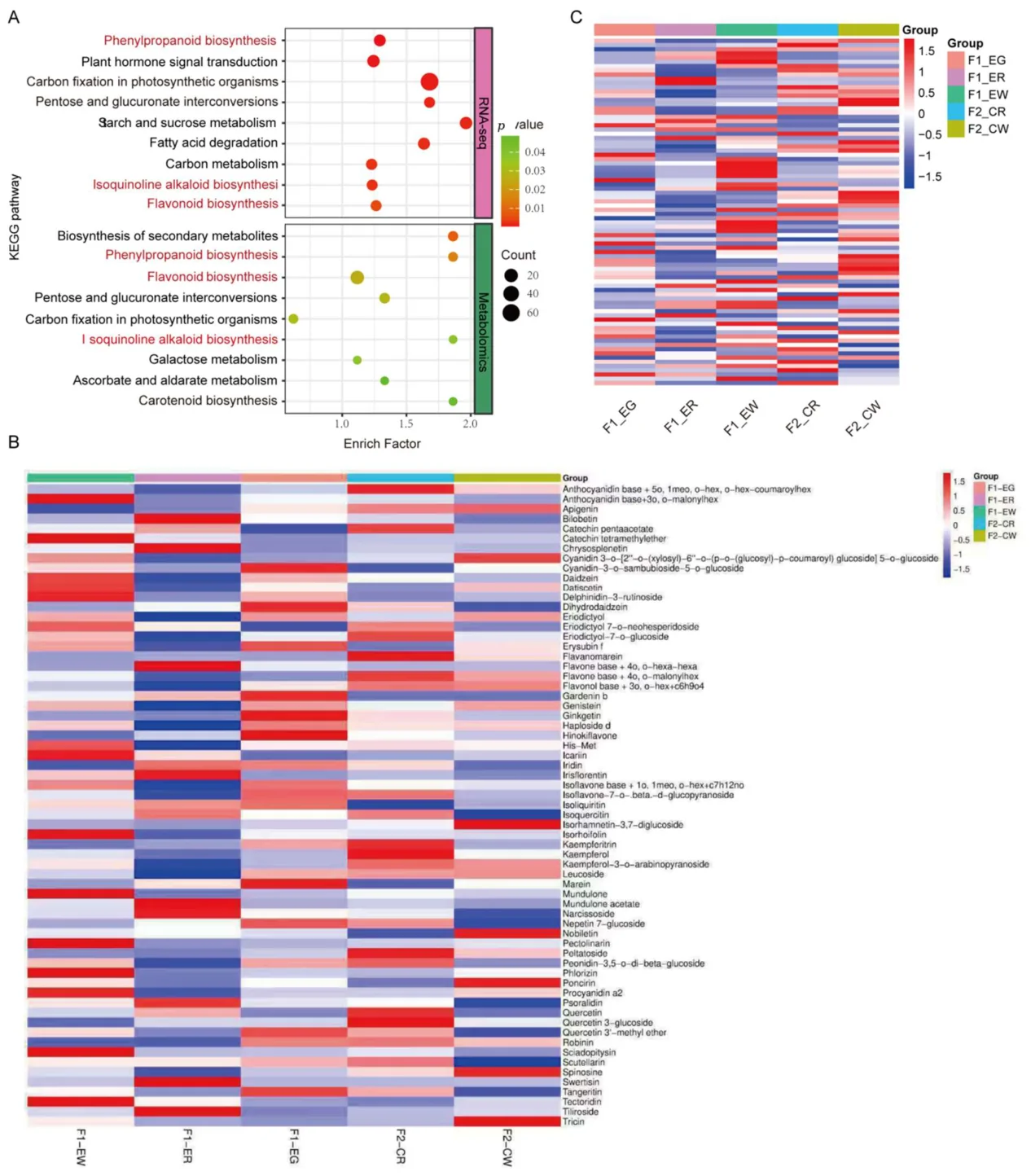
Integrated transcriptome–metabolome association analysis. (**A**) Kyoto Encyclopedia of Genes and Genomes (KEGG) enrichment of RNA-seq DEGs (upper panel) and metabolomic DAMs (lower panel). The *x*-axis is the enrichment factor; point size represents the number of annotated features, point color represents the *p*-value according to the displayed green-to-red scale, and red pathway labels identify the three pathways shared between the omics layers. (**B**) Heatmap of DAM abundance in the three highlighted pathways. (**C**) Heatmap of DEG expression in the same pathways. In (**B**,**C**), rows are metabolites or genes, columns are F1-EG, F1-ER, F1-EW, F2-CR, and F2-CW, red indicates relatively higher and blue relatively lower row-standardized values, and the top annotation colors identify groups as follows: F1-EG, salmon; F1-ER, purple; F1-EW, green; F2-CR, cyan; F2-CW, olive.

Joint pathway mapping identified 82 DEGs and 115 DAMs annotated to phenylpropanoid, flavonoid, and isoquinoline alkaloid biosynthesis (Figure 3A). Phenylpropanoid and flavonoid pathways have established roles in floral pigmentation, whereas isoquinoline alkaloid enrichment represents an association that may reflect broader secondary-metabolic differences among the sampled tissues. The present data do not demonstrate alkaloid–anthocyanin complex formation or a causal role for isoquinoline alkaloids in co-pigmentation.

Pearson correlation analysis (|r| > 0.8, *p* < 0.001) of DEGs and DAMs within these three pathways identified extensive and intricate gene–metabolite association networks, visualized using Cytoscape v3.9.1 (Figure S5). Among all detected metabolites, five flower color-associated metabolites were identified with the highest correlation coefficients (|r| > 0.8, FDR < 0.05): Anthocyanidin base+3-O,O-malonylhexoside, Cyanidin-3-O-sambubioside-5-O-glucoside, Delphinidin-3-rutinoside, Flavone base+4-O,O-hexosyl-hexoside, and Peonidin-3,5-O-di-β-glucoside. Together with their correlated structural and regulatory genes, these metabolites form the molecular signature of red flower color in *C. eburneum* × *C. insigne* F1 hybrids.

Based on the above analyses, differentially accumulated metabolites and differentially expressed genes were mapped to flower color-related pathways. The associations indicated that phenylalanine biosynthesis, phenylpropanoid metabolism, and flavonoid/anthocyanin biosynthesis were major candidate pathways associated with flower color variation. Key metabolites included phenylalanine, p-coumaric acid, caffeic acid, ferulic acid, and anthocyanin derivatives. The associated structural-gene abbreviations were hydroxycinnamoyl-CoA shikimate/quinate hydroxycinnamoyltransferase (*HCT*), p-coumarate 3-hydroxylase (*C3H*), 4-coumarate:CoA ligase (*4CL*), dihydroflavonol 4-reductase (*DFR*), anthocyanidin synthase/leucoanthocyanidin dioxygenase (*ANS*/*LDOX*), and UDP-glucose:flavonoid 3-O-glucosyltransferase (*UFGT*). These associations nominate pathway components but do not establish causal regulation.

### 3.5. qRT-PCR Validation of Key DEGs

**Figure 4:**
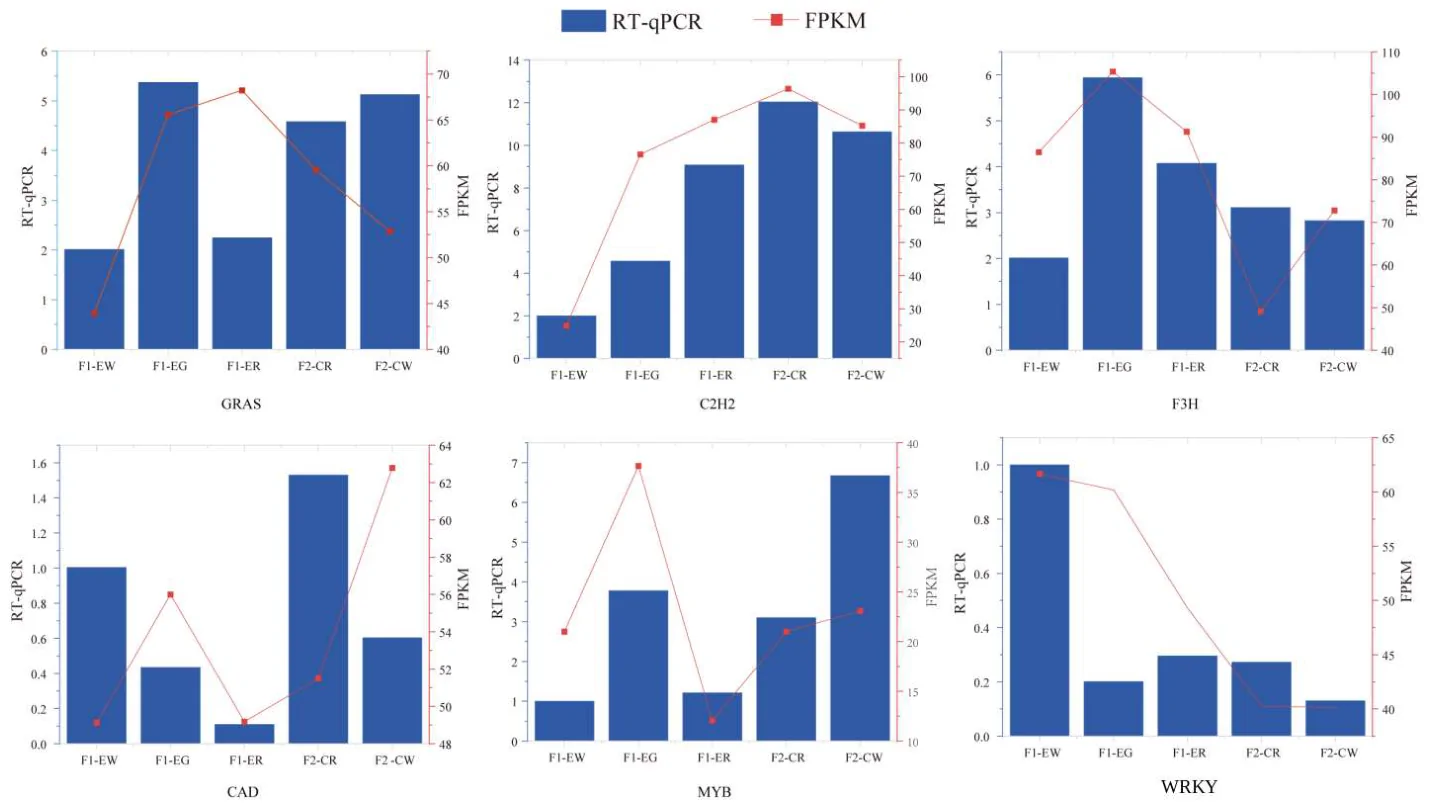
Descriptive comparison of archived qRT-PCR and RNA-seq profiles for six flower-color-associated candidate genes: *GRAS* (*JL003203*), *C2H2* (*JL016282*), *F3H* (*JL028702*), *CAD* (*JL007226*), *MYB* (*JL017958*), and *WRKY* (*JL010099*). Blue bars (left *y*-axis) show archived summarized qRT-PCR relative expression values calculated by the 2^−ΔΔCt^ method after normalization to *CymTIP41* and *CymEF1α*; the red line (right *y*-axis) connects the corresponding archived RNA-seq fragments per kilobase of transcript per million mapped reads (FPKM) values to show their across-category pattern. Categories are F1-EW, F1-EG, F1-ER, F2-CR, and F2-CW as defined in Table 1. Replicate-level qRT-PCR Ct records were not retained; therefore, error bars, biological-replicate points, and inferential statistics could not be calculated or displayed.

Six selected DEGs from phenylpropanoid/flavonoid biosynthesis and transcription-factor families were examined by qRT-PCR (Figure 4): *GRAS* (*JL003203*), *C2H2* (*JL016282*), *F3H* (*JL028702*), *CAD* (*JL007226*), *MYB* (*JL017958*), and *WRKY* (*JL010099*). The archived summarized qRT-PCR profiles and RNA-seq FPKM trends showed partial qualitative agreement across the five categories, but agreement was not uniform among genes. Because replicate-level qRT-PCR Ct values are unavailable, this comparison is descriptive and does not support an inferential claim of validation or reproducibility.

## 4. Discussion

Flower color is a major determinant of ornamental value in *Orchidaceae*. Here, integrated transcriptomic and metabolomic analyses were applied to F1 hybrids of *C. eburneum* × *C. insigne* to summarize molecular associations with five floral tissue/color categories. The results reinforce established phenylpropanoid and flavonoid/anthocyanin biology and identify candidate metabolites and co-expression modules. Isoquinoline alkaloid enrichment remains hypothesis-generating; the present data do not demonstrate cooperation or causality among three pathways.

Enrichment of phenylpropanoid and flavonoid/anthocyanin biosynthesis is consistent with established flower-pigmentation biology [34] and, by itself, is not novel. The contribution of this dataset lies instead in nominating specific metabolites and gene modules associated with the sampled *Cymbidium* F1 tissue/color categories. Isoquinoline alkaloid pathway enrichment should be interpreted cautiously because pathway-level enrichment does not establish pigment activity, physical co-pigmentation, or pathway causality.

The phenylpropanoid pathway supplies precursors for downstream flavonoid synthesis. Differential-expression and pathway analyses identified *PAL*, cinnamate 4-hydroxylase (*C4H*), and 4-coumarate:CoA ligase (*4CL*) among pigmentation-related candidates. This is consistent with the established role of phenylpropanoid metabolism in flavonoid biosynthesis [7,8]. However, the present WGCNA output alone does not establish these genes as module hubs or causal regulators; their proposed roles are based on pathway annotation and expression association and therefore require functional validation.

Flavonoid and anthocyanin biosynthesis has an established role in red floral pigmentation. In the present dataset, Cyanidin-3-O-sambubioside-5-O-glucoside, Delphinidin-3-rutinoside, and Peonidin-3,5-O-di-β-glucoside showed abundance patterns associated with red floral tissues. These compounds are therefore reported as candidate red-associated metabolites rather than as the sole or proven determinants of red coloration. Expression differences in *CHS*, *DFR*, *ANS*, and *UFGT* were also associated with the sampled tissue/color categories, but functional regulation was not tested experimentally.

Isoquinoline alkaloid biosynthesis was enriched in the integrated analysis; however, no targeted alkaloid quantification, structural confirmation, or anthocyanin–alkaloid complexation assay was performed. Accordingly, this result is treated as a hypothesis-generating association with broader secondary metabolism rather than evidence for alkaloid-mediated co-pigmentation. Direct chemical identification and in vitro or in vivo co-pigmentation experiments will be required to test this possibility.

The three red-associated anthocyanin derivatives differ in hydroxylation, methylation, and glycosylation state, chemical features that are known to influence anthocyanin color and stability [35]. However, the present widely targeted metabolomic data provide relative abundance and annotation-level evidence only. Structural identity, vacuolar pH effects, pigment complexation, and individual chromogenic contributions were not directly tested; consequently, implications for blue-flower engineering or pigment stabilization remain hypotheses for future targeted chemical and functional studies.

**Figure 5:**
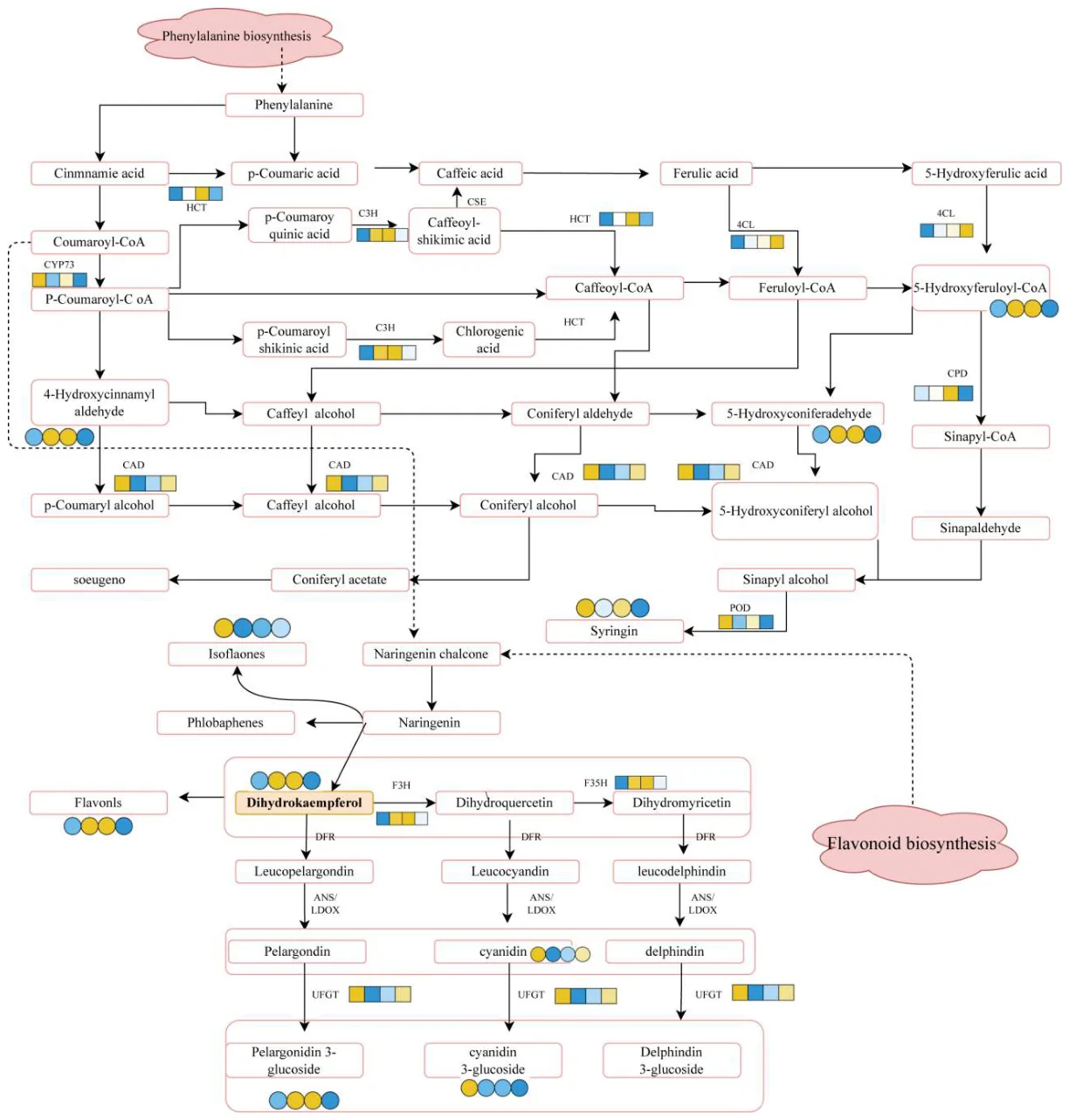
Author-constructed conceptual model of gene–metabolite associations relevant to orchid flower coloration. The model maps study DEGs and DAMs onto established KEGG phenylpropanoid biosynthesis (ko00940) and flavonoid biosynthesis (ko00941) relationships; no original KEGG artwork is reproduced. Rectangular five-cell symbols summarize gene-expression profiles and circular five-position symbols summarize metabolite-abundance profiles in the fixed left-to-right order F1-EW, F1-EG, F1-ER, F2-CR, and F2-CW. Yellow indicates relatively higher, blue relatively lower, and pale/white intermediate values within each feature. Solid arrows denote established direct biochemical steps; dashed arrows denote indirect or multistep pathway connections. Gene abbreviations are *HCT*, hydroxycinnamoyl-CoA shikimate/quinate hydroxycinnamoyltransferase; *C3H*, p-coumarate 3-hydroxylase; CSE, caffeoyl shikimate esterase; *4CL*, 4-coumarate:CoA ligase; *CAD*, cinnamyl alcohol dehydrogenase; CPD, 5-hydroxyconiferaldehyde dehydrogenase; POD, peroxidase; *F3H*, flavanone 3-hydroxylase; *F3′5′H*, flavonoid 3′,5′-hydroxylase; *DFR*, dihydroflavonol 4-reductase; *ANS*/*LDOX*, anthocyanidin synthase/leucoanthocyanidin dioxygenase; and *UFGT*, UDP-glucose:flavonoid 3-O-glucosyltransferase. This model serves to generate hypotheses rather than establish causal regulation.

Integrating these findings, we present in the Discussion a provisional model linking established phenylpropanoid/flavonoid metabolism with the gene–metabolite associations observed across the sampled tissue/color categories (Figure 5). The model summarizes the present associations and established biochemical steps; it is not presented as a newly discovered pathway or a validated causal regulatory architecture. Because tissue identity and color category are partly confounded and the network is based on 15 samples, the model serves only to organize testable candidates.

## 5. Conclusions

Integrated transcriptomic and metabolomic analyses of 15 samples identified 4891 non-redundant DEGs, 383 non-redundant DAMs, anthocyanin derivatives, pathway-level differences, and co-expression modules associated with five floral tissue/color categories in *C. eburneum* × *C. insigne* F1 hybrids. Phenylpropanoid and flavonoid/anthocyanin biosynthesis showed the clearest established relationship with pigmentation. Isoquinoline alkaloid enrichment remains an unvalidated association. The resulting candidate genes and metabolites require validation in tissue-matched samples and through independent genetic, biochemical, or functional experiments before causal roles in flower-color regulation can be assigned.

## Figures and Tables

**Table 1:** Sample codes, sampled floral tissues, flower colors, and RHS color-chart assignments used for transcriptomic and metabolomic analyses.

Sample ID	English Phenotype Description
F1-EG	*C. eburneum* × *C. insigne* F1: petals + sepals yellow (RHS 149D)
F1-ER	*C. eburneum* × *C. insigne* F1: labellum magenta with markings (RHS N66A)
F1-EW	*C. eburneum* × *C. insigne* F1: labellum white (RHS NN155)
F2-CR	*C. eburneum* × *C. insigne* F1: petals + sepals white background with red stripes (RHS 66C)
F2-CW	*C. eburneum* × *C. insigne* F1: petals + sepals white (RHS NN155)

Note: Cell background colors schematically represent the observed flower-color categories corresponding to the listed RHS Colour Chart codes.

**Table 2:** Gene names, complete gene identifiers, primer sequences, and amplicon sizes used for qRT-PCR validation.

Gene Abbreviation and Full Name	Gene ID	Forward Primer (5′–3′)	Reverse Primer (5′–3′)	Product (bp)
*GRAS* (GRAS family transcription factor)	*JL003203*	CCGTGAACTTGGCTTTCCAG	AGGCTCTTCACGAACCTCAG	94
*C2H2* (Cys2-His2-type zinc finger transcription factor)	*JL016282*	GTTGTCCGGTTTGTGGAGTG	GATCCAACACCACCGCTTTC	144
*F3H* (flavanone 3-hydroxylase)	*JL028702*	CAGAACGACGGTGAACAAGG	AAACTTGGCCGGATTCTTGC	121
*CAD* (cinnamyl alcohol dehydrogenase)	*JL007226*	CTTTGCCTCTGCCATGATCG	AGCCCGAAGTTCTTCAGAGG	129
*MYB* (MYB family transcription factor)	*JL017958*	CATCTCCAAGCACTGTTAGGC	GCTGGCCCTTCTTTTGAGAG	174
*WRKY* (WRKY family transcription factor)	*JL010099*	AGGAAGATGCTGCCTACCTG	TGCGTGCATCGGTAGTAGG	152

**Table 3:** WGCNA candidate genes associated with red, yellow, and white floral tissue/color categories.

Associated Flower Color (WGCNA Module)	Candidate Gene ID	Functional Annotation
Red (darkseagreen4)	*JL016799*	U1 small nuclear ribonucleoprotein 70 kDa
Red (darkseagreen4)	*JL020957*	Zinc finger CCCH domain-containing protein 5
Red (darkseagreen4)	*JL000049*	CCR4-NOT transcription complex subunit 1
Red (darkseagreen4)	*JL010156*	Phenolic glucoside malonyltransferase 2
Red (darkseagreen4)	*JL017507*	Cytochrome P450 81Q32
Red (darkseagreen4)	*JL027414*	Uncharacterized protein
Red (darkseagreen4)	*JL007156*	Glutamate--tRNA ligase, chloroplastic/mitochondrial
Red (darkseagreen4)	*JL000779*	Uncharacterized protein
Red (darkseagreen4)	*JL010027*	Zinc finger CCCH domain-containing protein 20
Red (darkseagreen4)	*JL014470*	Uncharacterized protein
Yellow (lightgreen)	*JL020768*	Uncharacterized protein
Yellow (lightgreen)	*JL010806*	Phosphoribulokinase, chloroplastic
Yellow (lightgreen)	*JL010805*	Uncharacterized protein
Yellow (lightgreen)	*JL001469*	Carbonic anhydrase, chloroplastic
Yellow (lightgreen)	*JL010486*	Uncharacterized protein
Yellow (lightgreen)	*JL016806*	Rhodanese-like domain-containing protein 4, chloroplastic
Yellow (lightgreen)	*JL009410*	Zerumbone synthase
Yellow (lightgreen)	*JL002831*	Probable nucleoredoxin 2
Yellow (lightgreen)	*JL012705*	Uncharacterized protein
Yellow (lightgreen)	*JL011521*	Uncharacterized protein
White (plum1)	*JL015236*	Chlorophyll a-b binding protein 40, chloroplastic
White (plum1)	*JL019281*	DNA polymerase alpha subunit B
White (plum1)	*JL001887*	Uncharacterized protein
White (plum1)	*JL020762*	Protein ecdysoneless homolog
White (plum1)	*JL003788*	Uncharacterized protein
White (plum1)	*JL009407*	CWF19-like protein 2
White (plum1)	*JL016580*	Uncharacterized protein
White (plum1)	*JL020046*	Ferredoxin-2, chloroplastic
White (plum1)	*JL019159*	Multiple C2 domain and transmembrane region protein 7
White (plum1)	*JL004250*	ER membrane protein complex subunit 7 homolog

Note: The listed genes met the prespecified module-membership and gene-significance thresholds. WGCNA associations identify co-expression candidates and do not by themselves demonstrate causal regulation of flower color.

## Data Availability

The raw RNA-seq data generated in this study are publicly available in the NCBI Sequence Read Archive under BioProject accession PRJNA1338804. The *Cymbidium ensifolium* reference genome used for read alignment is publicly available in the Genome Warehouse (GWH), National Genomics Data Center, under assembly accession GWHBCII00000000, BioProject PRJCA005355, and BioSample SAMC383359 (https://ngdc.cncb.ac.cn/gwh/Assembly/20686/show, accessed on 20 August 2026). The metabolomics data and processed data tables supporting the findings are available from the corresponding author upon reasonable request. Figures S1–S5 and their legends are provided with the article as Supplementary Materials.

## Supplementary Materials

The following supporting information can be downloaded at https://www.mdpi.com/article/10.3390/genes17091003/s1. Figures S1–S5 and their complete, self-contained legends are provided in the accompanying supplementary file. Figure S1 shows the parental and offspring flower phenotypes and sample-category codes; Figures S2–S4 show pathway-enrichment analyses; and Figure S5 shows gene–metabolite association networks.

## References

[1] Christenhusz M.J.M., Byng J.W. (2016). The number of known plant species in the world and its annual increase. Phytotaxa.

[2] Givnish T.J., Spalink D., Ames M., Lyon S.P., Hunter S.J., Ojeda A., Sytsma K.J. (2015). Orchid phylogenomics and multiple drivers of their extraordinary diversification. Proc. R. Soc. B Biol. Sci..

[3] Grotewold E. (2006). The genetics and biochemistry of floral pigments. Annu. Rev. Plant Biol..

[4] Tanaka Y., Sasaki N., Ohmiya A. (2008). Biosynthesis of plant pigments: Anthocyanins, betalains and carotenoids. Plant J..

[5] Fiehn O. (2002). Metabolomics—The link between genotypes and phenotypes. Plant Mol. Biol..

[6] Ohmiya A., Taniguchi S., Kondo T. (2014). Identification of genes associated with chlorophyll accumulation in flower petals. PLoS ONE.

[7] Holton T.A., Cornish E.C. (1995). Genetics and biochemistry of anthocyanin biosynthesis. Plant Cell.

[8] Winkel-Shirley B. (2001). Flavonoid biosynthesis. A colorful model for genetics, biochemistry, cell biology, and biotechnology. Plant Physiol..

[9] Griesbach R.J. (2005). Biochemistry and genetics of flower color. Plant Breed. Rev..

[10] Gomez C., Terrier N., Goupil P., Cheynier V., Moutounet M. (2011). In vivo grapevine anthocyanin transport involves vesicle-mediated trafficking and the contribution of anthoMATE transporters. Plant Cell.

[11] Zhao J., Dixon R.A. (2010). The ‘ins’ and ‘outs’ of flavonoid transport. Trends Plant Sci..

[12] Li B., Zheng H., Li S., Gong Q., Palfalvi G., Li Z. (2017). Molecular cloning and functional analysis of the anthocyanin transporter gene GmAM1 in *Gentiana macrophylla*. Front. Plant Sci..

[13] Ramsay N.A., Glover B.J. (2005). *MYB*-bHLH-WD40 protein complex and the evolution of cellular diversity. Trends Plant Sci..

[14] Quattrocchio F., Verweij W., Kroon A., Spelt C., Mol J., Koes R. (2006). PH4 of petunia is an R2R3 *MYB* protein that activates vacuolar acidification through interactions with basic-helix-loop-helix transcription factors of the anthocyanin pathway. Plant Cell.

[15] Jia N., Liu X., Guan L., Li C., Zhang H. (2022). The *Rosa rugosa RhMYB114*-*RhbHLH42* complex promotes anthocyanin biosynthesis by activating the expression of *RhDFR* and *RhANS*. Hortic. Res..

[16] Mol J., Grotewold E., Koes R. (1998). How genes paint flowers and seeds. Trends Plant Sci..

[17] Hsu C.-C., Chen Y.-Y., Tsai W.-C., Chen W.-H., Chen H.-H. (2015). Three R2R3-*MYB* transcription factors regulate distinct floral pigmentation patterning in *Phalaenopsis* spp.. Plant Physiol..

[18] Li Y., Zhao X., Zhang M.M., He X., Huang Y., Ahmad S., Lan S. (2022). Selection and validation of reference genes for quantitative real-time PCR analysis in *Cymbidium* hybrids. Plant Methods.

[19] Liu D.K., Tu X.D., Li L.Q., Chen J.Y., Ma X.K., Liu Z.J. (2020). Interspecific hybridization and characterization of hybrids between *Cymbidium insigne* and *C. eburneum*. Sci. Hortic..

[20] Love M.I., Huber W., Anders S. (2014). Moderated estimation of fold change and dispersion for RNA-seq data with DESeq2. Genome Biol..

[21] Gu Z., Gu L., Zhao Y., Chen X. (2019). Complex evolution of the chalcone synthase gene family in the genus *Paeonia* (*Paeonia*ceae). Sci. Rep..

[22] Yamagishi M., Shimoyamada Y., Nakatsuka T., Masuda K. (2010). Two R2R3-*MYB* genes, homologs of petunia AN2, regulate anthocyanin biosyntheses in flower tepals, tepal spots and leaves of Asiatic hybrid lily. Plant Cell Physiol..

[23] Thill J., Miosic S., Ahmed R., Schlangen K., Muster G., Stich K., Halbwirth H. (2012). ‘Le rouge et le noir’: A decline in flavone formation correlates with the rare color of black dahlia (*Dahlia variabilis* hort.) flowers. BMC Plant Biol..

[24] Petroni K., Tonelli C. (2011). Recent advances on the regulation of anthocyanin synthesis in reproductive organs. Plant Sci..

[25] Han Y., Cui Y., Chen Y., Rao D., Wu E., Gan R., Tian M. (2023). Identification and genetic diversity analysis of hybrid progenies from *Cymbidium eburneum* x *Cymbidium insigne* by SSR. Chin. J. Trop. Crops.

[26] Weckwerth W. (2011). Green systems biology—From single genomes, proteomes and metabolomes to ecosystems research and biotechnology. J. Proteom..

[27] Chen S., Zhou Y., Chen Y., Gu J. (2018). fastp: An ultra-fast all-in-one FASTQ preprocessor. Bioinformatics.

[28] Ai Y., Li Z., Sun W.-H., Chen J., Zhang D., Ma L., Zhang Q.-H., Chen M.-K., Zheng Q.-D., Liu J.-F. (2021). The *Cymbidium* genome reveals the evolution of unique morphological traits. Hortic. Res..

[29] Dobin A., Davis C.A., Schlesinger F., Drenkow J., Zaleski C., Jha S., Batut P., Chaisson M., Gingeras T.R. (2013). STAR: Ultrafast universal RNA-seq aligner. Bioinformatics.

[30] Pertea M., Pertea G.M., Antonescu C.M., Chang T.-C., Mendell J.T., Salzberg S.L. (2015). StringTie enables improved reconstruction of a transcriptome from RNA-seq reads. Nat. Biotechnol..

[31] Yu G., Wang L.-G., Han Y., He Q.-Y. (2012). clusterProfiler: An R package for comparing biological themes among gene clusters. OMICS.

[32] Langfelder P., Horvath S. (2008). WGCNA: An R package for weighted correlation network analysis. BMC Bioinform..

[33] Shannon P., Markiel A., Ozier O., Baliga N.S., Wang J.T., Ramage D., Ideker T. (2003). Cytoscape: A software environment for integrated models of biomolecular interaction networks. Genome Res..

[34] Koes R., Verweij W., Quattrocchio F. (2005). Flavonoids: A colorful model for the regulation and evolution of biochemical pathways. Trends Plant Sci..

[35] Du H., Wu J., Ji K.-X., Zeng Q.-Y., Bhuiya M.W., Su S., Shu Q.-Y., Ren H.-X., Liu Z.-A., Wang L.S. (2015). Methylation mediated by an anthocyanin O-methyltransferase is involved in purple flower coloration in *Paeonia*. J. Exp. Bot..

