# Supplementary material for: Integrated Multi-Omics Analysis Reveals Key Metabolites and Regulatory Networks Underlying Flower Color Variation in F1 Hybrids of Cymbidium eburneum × Cymbidium insigne
Source: Genes (Basel). 2026 Aug 26;17(9):1003. doi: 10.3390/genes17091003 (PMC13606605; doi:10.3390/genes17091003)
Supplement: Supplementary file 1 [file genes-17-01003-s001.zip › genes-4469911-supplementary.pdf]

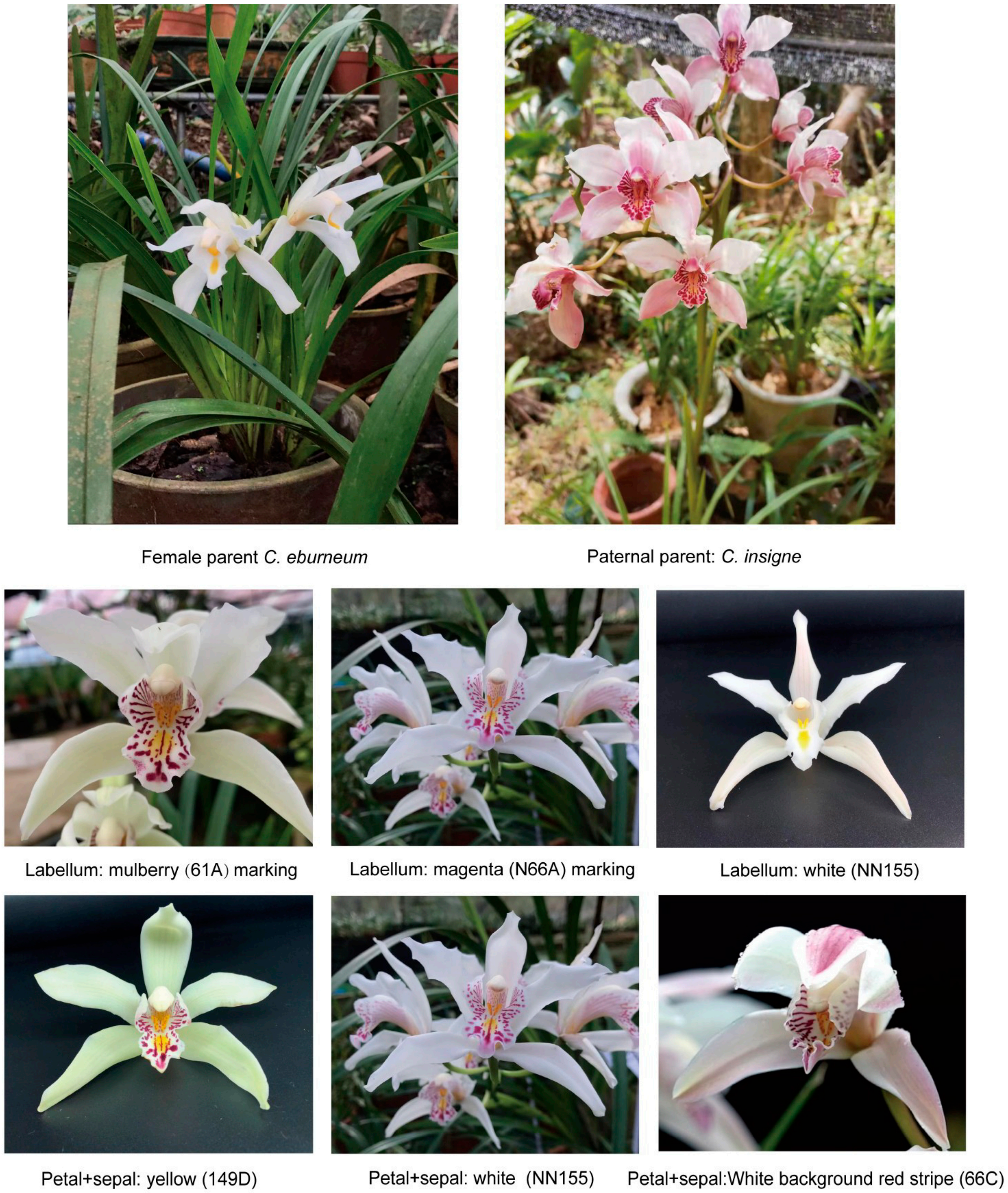

Supplementary Figure S1. Representative flower colors of the parental species and sampled F1 offspring. *Cymbidium eburneum* and *Cymbidium insigne* are the parental species. Sample-category codes are: F1-EG, yellow petals and sepals (RHS 149D); F1-ER, red/magenta labellum (RHS N66A); F1-EW, white labellum (RHS NN155); F2-CR, white-background red-striped petals and sepals (RHS 66C); and F2-CW, white petals and sepals (RHS NN155).

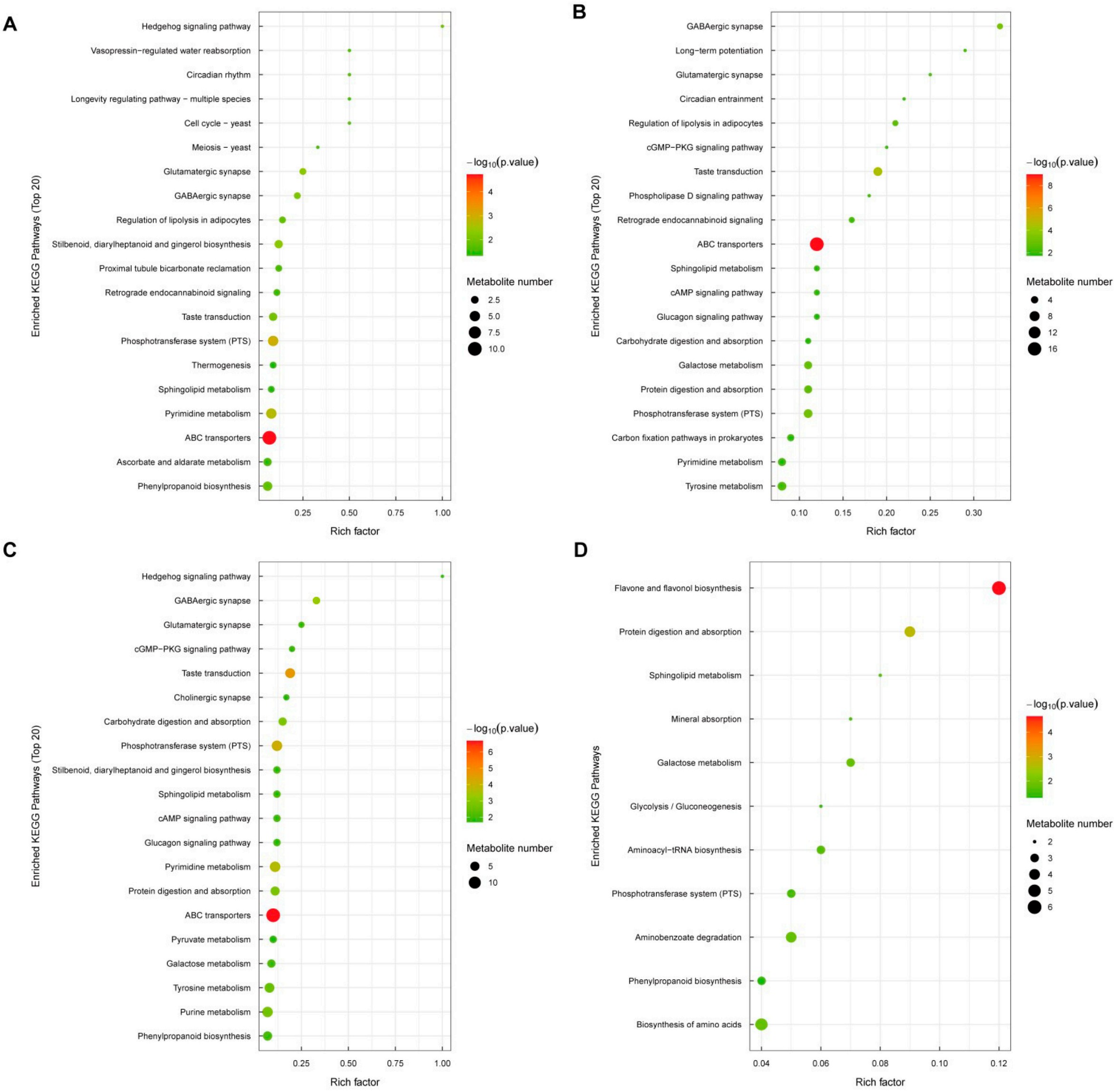

Supplementary Figure S2. Kyoto Encyclopedia of Genes and Genomes (KEGG) pathway enrichment of differentially accumulated metabolites (DAMs) in four comparisons: (**A**) F1-EG versus F1-EW; (**B**) F1-ER versus F1-EG; (**C**) F1-ER versus F1-EW; and (**D**) F2-CR versus F2-CW. The x-axis shows the rich factor; point size represents the number of DAMs assigned to a pathway, and point color represents enrichment significance according to the displayed scale.

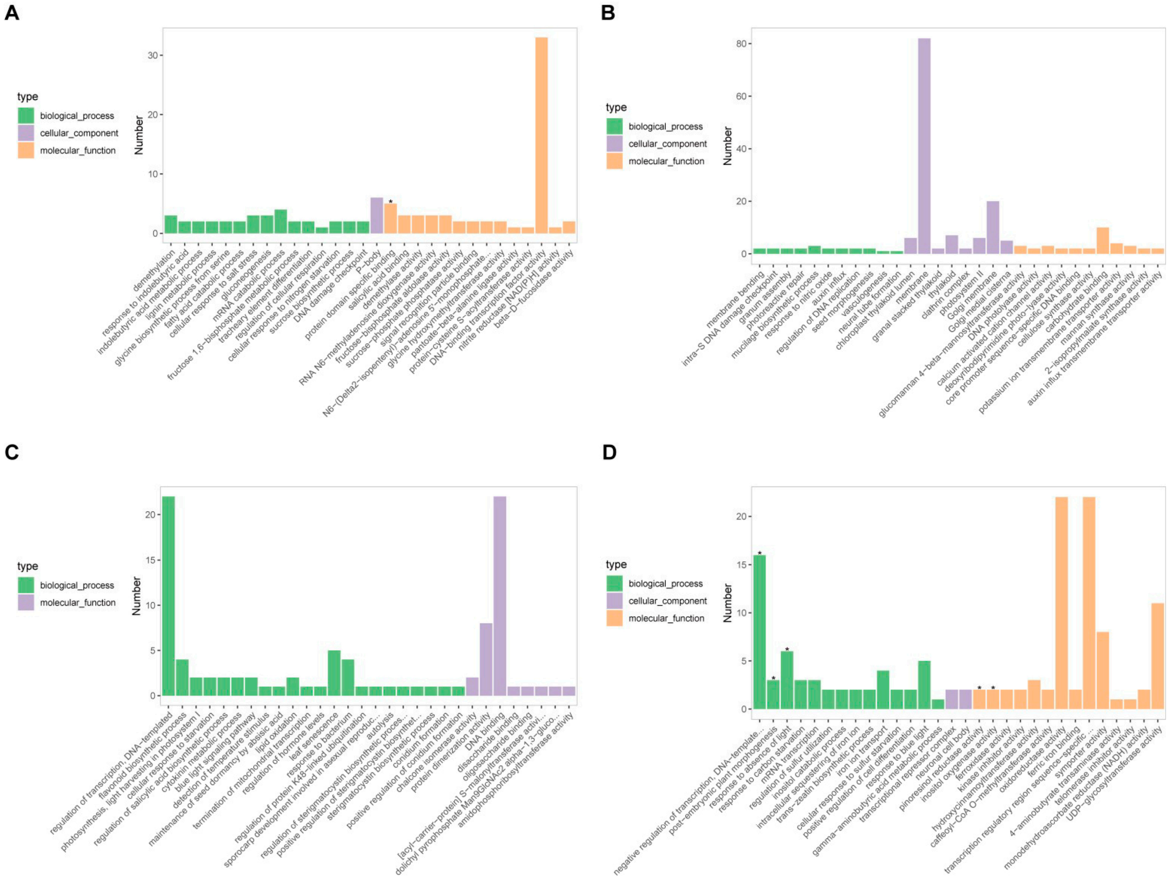

Supplementary Figure S3. Gene Ontology (GO) enrichment of differentially expressed genes (DEGs) in four comparisons: (**A**) F1-EG versus F1-EW; (**B**) F1-ER versus F1-EG; (**C**) F1-ER versus F1-EW; and (**D**) F2-CR versus F2-CW. Terms are grouped into biological process, cellular component, and molecular function. Bar length represents the number of annotated DEGs, and bar color represents enrichment significance according to the displayed scale.

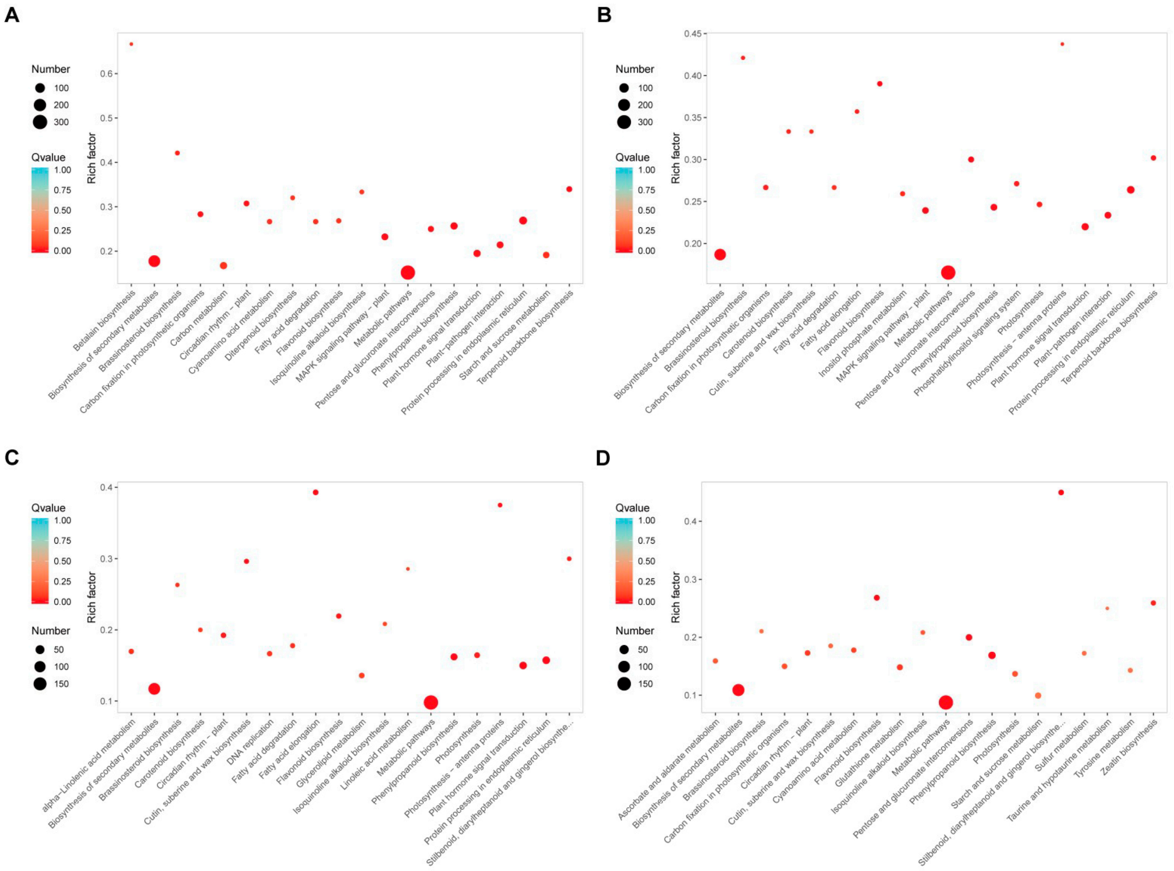

Supplementary Figure S4. Kyoto Encyclopedia of Genes and Genomes (KEGG) pathway enrichment of differentially expressed genes (DEGs) in four comparisons: (**A**) F1-EG versus F1-EW; (**B**) F1-ER versus F1-EG; (**C**) F1-ER versus F1-EW; and (**D**) F2-CR versus F2-CW. The x-axis shows the rich factor; point size represents the number of DEGs assigned to a pathway, and point color represents enrichment significance according to the displayed scale.

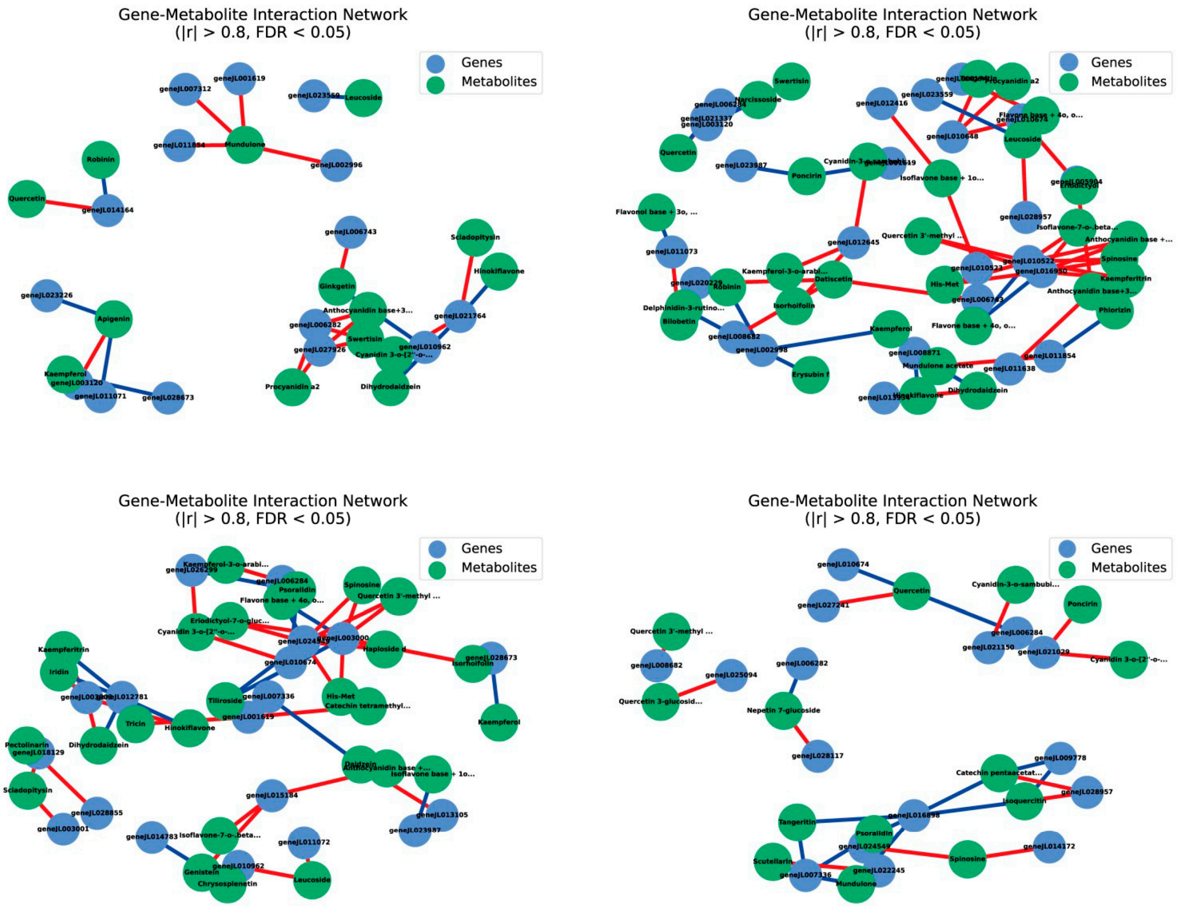

Supplementary Figure S5. Gene–metabolite association networks for four comparisons: (**A**) F1-EG versus F1-EW; (**B**) F1-ER versus F1-EG; (**C**) F1-ER versus F1-EW; and (**D**) F2-CR versus F2-CW. Blue nodes are genes and green nodes are metabolites. Red edges indicate positive and blue edges negative Pearson correlations. Only associations satisfying $|r| > 0.8$ and false-discovery-rate-adjusted $p < 0.05$ are shown.
